# Perceived structural vulnerabilities among detained noncitizen immigrants in Minnesota

**DOI:** 10.1371/journal.pone.0252232

**Published:** 2021-06-09

**Authors:** Kazumi Tsuchiya, Olivia Toles, Christopher Levesque, Kimberly Horner, Eric Ryu, Linus Chan, Jack DeWaard

**Affiliations:** 1 Minnesota Population Center, University of Minnesota, Minneapolis, Minnesota, United States of America; 2 College of Biological Sciences, University of Minnesota, Minneapolis, Minnesota, United States of America; 3 Department of Sociology, University of Minnesota, Minneapolis, Minnesota, United States of America; 4 Humphrey School of Public Affairs, University of Minnesota, Minneapolis, Minnesota, United States of America; 5 University of Minnesota Law School, University of Minnesota, Minneapolis, Minnesota, United States of America; Drexel University, UNITED STATES

## Abstract

Across several decades there has been an unprecedented increase in immigration enforcement including detention and deportation. Immigration detention profoundly impacts those experiencing detention and their family members. An emerging area of research has found that immigrants experience a number of challenges which constrain and limit their decisions, choices, and options for security and integration in the United States due to social, political and structural determinants. These determinants lead to greater structural vulnerabilities among immigrants. The purpose of the current study was to illuminate the perceived vulnerabilities of detained noncitizen immigrants as they are raised and described while attending case hearings at the Bloomington, Minnesota immigration court. Through conducting a thematic analysis of notes derived from third party immigration court observers, three areas of perceived vulnerability were identified. These perceived vulnerabilities include 1) migration and motivations to migrate, 2) structural vulnerabilities (e.g., discrimination, financial insecurity, social ties and family support, stable or fixed residence, English language proficiency, health and mental health) in the US, and 3) challenges in navigating immigration detention. These findings demonstrate that noncitizen immigrants who are undergoing immigration detention are experiencing multiple intersecting vulnerabilities which profoundly impact their lives. Collaborative efforts across sectors are needed to work towards comprehensive immigration reforms including both short-term and long-term solutions to address pressing issues for noncitizens undergoing immigration detention.

## Introduction

Across several decades there has been an unprecedented increase in immigration enforcement against noncitizen immigrants, including detainment and deportation by the US government [1]. Noncitizens comprise about half of all immigrants in the US, including legal permanent residents (27%), temporary visa status holders (5%) and undocumented (23%) immigrants [2]. Notably, the daily population of detained immigrants has increased seven-fold, from 6,785 in 1994 to 48,850 in 2019 [3, 4]. Record numbers of deportations have been noted within the past decade, with 409,000 in 2012 and 267,278 in 2019 [4, 5]. Previous research has found that immigrants undergoing detention experience vulnerabilities spanning across multiple systems that permeate different aspects of their life (e.g., economic, health) and extend to their families [1].

Immigrants experience a number of challenges and risks stemming from upstream social-structural factors which constrain and limit their options for security and integration in the US [6, 7]. These inequities are a byproduct of the hierarchical social structures and power relations enforced through policies, practices, and laws concerning an individual’s legal status and other attributes [6, 7]. Adversities tend to co-occur among those experiencing structural vulnerabilities [8]. However, there has been limited understanding of these vulnerabilities across multiple systems, including immigration detention among detained noncitizens, and how these vulnerabilities may intersect and contribute to further marginalization. Thus, the purpose of the current study is to expand our understanding of perceived structural vulnerabilities experienced by noncitizens undergoing detention.

### Structural vulnerability

Vulnerability is a multidimensional construct, which comes in many different forms [8]. The concept of structural vulnerability has its roots in medical anthropology and has gained prominence within the last several decades [9]. Structural vulnerability recognizes and highlights how upstream macro social structures contribute to suffering, distress, and health, and it has been broadly defined in the literature among marginalized groups [6, 9]. The fields of public health and medicine have also recognized these mechanisms of upstream macro-level factors in contributing to health inequities and refer to them as social determinants of health [10, 11].

According to the Theory of Fundamental Causes, structurally rooted disadvantages or fundamental causes involve access to resources, which have adverse implications for health and treatment of disease [11]. These resources include money, knowledge, power, prestige, and interpersonal resources of social support, which may in turn, mitigate the risk of disease while increasing access to medical treatment. The Theory of Fundamental Causes provides an organizing framework to understand sources of structural vulnerabilities among marginalized populations, particularly noncitizen immigrants.

To measure structural vulnerability, Bourgois and colleagues [6] created a structural vulnerability assessment tool to be used by health care providers which incorporates potential sources of vulnerability. This tool identified eight domains of vulnerability including financial security, residence, risk environments, food access, social network, legal status, education, and discrimination. The purpose of this tool was to promote understanding of how social conditions may influence patients’ health in medical settings. Cartwright [12] examined sources of vulnerability among Latino agricultural workers who were either legal permanent residents or undocumented immigrants. They found structural vulnerabilities arise across three general domains: 1) difficulties, delays, and denials stemming from the US immigration system, 2) inability to obtain steady, safe, and livable wages, and 3) challenges in accessing adequate health care (e.g., health insurance, medical care). These domains identified by Bourgois [6] and later by Cartwright [12] may also be sources of vulnerability for noncitizen immigrants in the US.

Migration is a source of vulnerability and a determinant of health [13, 14], coupled with heightened vulnerability due to the resulting challenges and conditions of living in the US. Legal and citizenship status is considered to be a fundamental cause of health among immigrants, acting as a form of social exclusion in determining access to resources and affecting health through multiple pathways [15]. Holmes [9] found that, among migrant farm workers, the characteristics and depth of an individual’s vulnerability changed depending on their position in the agricultural labor structure and the global economy more generally, as well as due to one’s legal status and ethnicity. Legal and citizenship status in the US determines belonging and exclusion through access to rights (e.g., voting) and social and economic resources including income and education [12, 16]. Scholars have described legal statuses as ‘scattered along multidimensional space’ and shaped by law as well as social realities [17]. Noncitizens may include legal permanent residents, temporary visa status holders, as well as undocumented immigrants–all experiencing differing degrees of access to social capital, education, and public assistance [12, 16]. Yoshikawa [18] found undocumented status to be a marker of marginalization evidenced by lower wages and social capital, and poor working conditions among recent Mexican and Chinese immigrants. In support of these findings, noncitizens are more likely to live in poverty compared to US born or naturalized citizens [19]. Noncitizens may be experiencing greater challenges and vulnerabilities, which has adverse implications for their successful integration and health.

### Compounding structural vulnerabilities in the immigration detention system

Noncitizens not only experience greater vulnerabilities from the broader societal structures [15, 16] but they are also vulnerable to deportation due to the lack of legal protections and access to resources [1, 20]. The structure of the immigration regime compounds the vulnerabilities already faced by noncitizens [1]. Emerging research demonstrates that immigration judges presiding over immigration courts function as immigration court actors, often considering the background of the detainee in deciding bond and removal outcomes [21–23]. Asad [21] further argues that multiple dimensions of exclusion are encoded into immigration law, including nuances in judicial decision-making, which contribute to variation in outcomes for noncitizen immigrants undergoing immigration proceedings, as further detailed in this section. Evidence suggests that detainees may have limited access to economic and interpersonal resources, as they are more likely to live in poverty, have low levels of education and English language proficiency, as well as limited social ties in the US, which contributes to negative outcomes for their release from detention [1, 24, 25]. According to the Theory of Fundamental Causes, these resources contribute to detrimental outcomes for health. In the context of immigration court proceedings, limited access to economic and interpersonal resources contributes to heightened vulnerability and greater marginalization for noncitizens undergoing immigration detention [1].

Emerging research has examined linkages between individuals who have been detained and deported under immigration law and compared these to those who are incarcerated under criminal law [1]. A key distinction between the two is that immigration law is considered to be civil law, meaning immigration detention should be administrative and nonpunitive. However, it is critical to note that this distinction has become blurred with the criminalization of noncitizens and the convergence of immigration law with criminal law or “crimmigration” [26]. In contrast to US citizens, detained noncitizens are not provided with constitutional protections, which include the Sixth Amendment right to counsel [27]. Although noncitizens may obtain and must pay for their own counsel, many face immigration proceedings without representation, which often has deleterious consequences for immigration proceedings and bond outcomes [23]. A study conducted in 2015 found that those with representation (in the form of an attorney), compared to those without representation (or *pro se*), had significantly better outcomes including obtaining bond and relief from removal [28]. These different outcomes may be attributed to the biases of immigration judges, who may view a detainee with an attorney at a bond hearing as a “worthy opponent”–someone deemed as deserving of attention, respect, and opportunity by the judge [23]. Additionally, having representation may signal to the immigration judge that the detainee is committed to and invested in the legal process and are less likely to be a flight risk, where they are likely to flee or abscond, than detainees without representation. Further, empirical research has demonstrated that immigration judges use individual case characteristics as proxies for how they determine whether noncitizens are “American” and if they are “deserving” to stay or alternatively, if they are considered to be a “danger to society” [22, 29]. Therefore, noncitizens may not experience structural vulnerability uniformly, as their vulnerabilities may be heightened among those without representation (or *pro se* detainees) during immigration court proceedings.

Many noncitizens are held in immigration detention for extended periods of time due to the large volume and backlog of cases [23]. There are no limits on the length of time that they can be detained and for some, they may be in detention for the entirety of their removal proceedings [1]. According to the Transactional Records Access Clearinghouse (TRAC) data [30], noncitizens were detained from six months to more than three years. Golash-Boza [31] found that among noncitizens who were held in immigration detention and in prison, being held in detention was considered to be worse due to the uncertainty of their release, along with the lack of programming and the ability to purchase food. Furthermore, the uncertainty of release generates substantial stress, especially for those who have been detained for lengthy periods of time [32]. Based on these findings, undergoing immigration detention in and of itself is another realm of vulnerability.

Detention can also have severe financial and health consequences for detainees and their families [1]. Patler [33] conducted a survey among 562 immigrants who were detained in Southern California for an average of nine months and found that the majority had lived in the US for at least 20 years and had either a spouse or child who was a US citizen or lawful permanent resident (69%). Family members of detained immigrants experienced difficulties paying rent or utilities (63%), medical expenses (42%), and food (37%). The immigration bond system further exacerbates and reproduces inequalities across family members [1]. These challenges are heightened and prolonged by a system that requires bond to be paid in full before a detainee can be released, yet many detained immigrants received bond amounts that were either above their financial means or were denied bond [1, 23]. Additionally, Pinedo and Valdez [34] found that Latinx US citizens who either had a family member or knew someone who was detained or deported reported worse mental health compared to whites or Latinx citizens who did not know anyone who was detained or deported. Another study by Zayas and colleagues [35] found that US citizen children who had parents who were detained or deported had worse mental health, compared to citizen children with undocumented parents who did not experience detention or deportation. These findings suggest that noncitizen immigrants undergoing detention and their families are experiencing multiple vulnerabilities under extreme immigration surveillance.

### The current study

In order to develop a deeper and more nuanced understanding of the intersecting vulnerabilities and challenges of noncitizens experiencing detention, this study draws on the evidence presented at immigration court hearings as well as the judicial decisions issued at these hearings. Immigration court hearings have profound implications for the lives of detained noncitizens. Additionally, issues that arise during these hearings provide context to better understand potential sources of perceived vulnerabilities. The current study documents and examines multiple dimensions of perceived vulnerabilities experienced by detained immigrants attending hearings in the Bloomington, Minnesota immigration court. This court primarily hears cases for individuals apprehended in Minnesota, South Dakota, and North Dakota. The purpose of the current study is to 1) to inventory perceived vulnerabilities of noncitizen immigrants as they are raised and described during immigration court proceedings and 2) to describe vulnerabilities which are specific to immigration detention.

## Methods

### Data

Data for this study were obtained from the Human Rights Defender Project (HRDP) and were collected from July 2018 to June 2019. The HRDP, which was launched in the Twin Cities area (Minneapolis, St. Paul) in Minnesota after the 2016 presidential election, is a collaboration across multiple partners, including the Advocates for Human Rights, Robins Kaplan LLP, and the University of Minnesota Law School’s James H. Binger Center for New Americans. The primary goals for the HRDP are two-fold, 1) to provide pro bono representation to detained immigrants, and 2) to scaffold an opportunity for community volunteers to conduct public third-party immigration court observations documenting the courtroom experiences of noncitizens in detention undergoing immigration proceedings.

As a volunteer-driven project, HRDP court observers were recruited from the University of Minnesota, faith-based organizations, and local human rights organizations, all within the Twin Cities area in Minnesota. Background and demographic information from the HRDP observers were not collected for this project; however, the Advocates for Human Rights report provides further details of the volunteers’ background information [25].

HRDP observers attended master calendar hearings held at the Bloomington, Minnesota immigration court. Master calendar hearings are preliminary hearings to begin the process of removal for immigrants in the US. Volunteers completed court observation forms that were created by the HRDP project organizers and volunteers. This form included a total of 48 questions that were a mixture of open- and closed-ended questions covering the topics of bond outcomes, legal representation, mental health conditions, criminal history, judge and attorney engagement, and language and interpreter usage. Eleven open-ended questions were included on the form as opportunities to capture further details to some of the closed-ended questions regarding bond outcomes, criminal history, interpretation challenges, mental health conditions, and any other additional concerns that were mentioned during the hearing. We focus on responses to these eleven open-ended questions in this paper. Please refer to the report by the Advocates for Human Rights [25] for the sample questionnaire and further details on the open-ended questions. Our data represent perceptions of structural vulnerabilities from detailed notes summarized by the HRDP court observers. Although we were unable to obtain direct accounts from the detainees regarding their vulnerabilities, these notes nonetheless allow us to obtain a snapshot of some of the perceived vulnerabilities experienced by detained noncitizens.

The data for the current study were collected from third party observers of immigration court hearings. Thus, it was determined that the data collected from the public hearings and used for the current study did not require IRB approval. Nevertheless, all unique identifiers (name, place of birth, etc.) were removed from the data and data were analyzed anonymously.

### Sample

**Fig 1:**
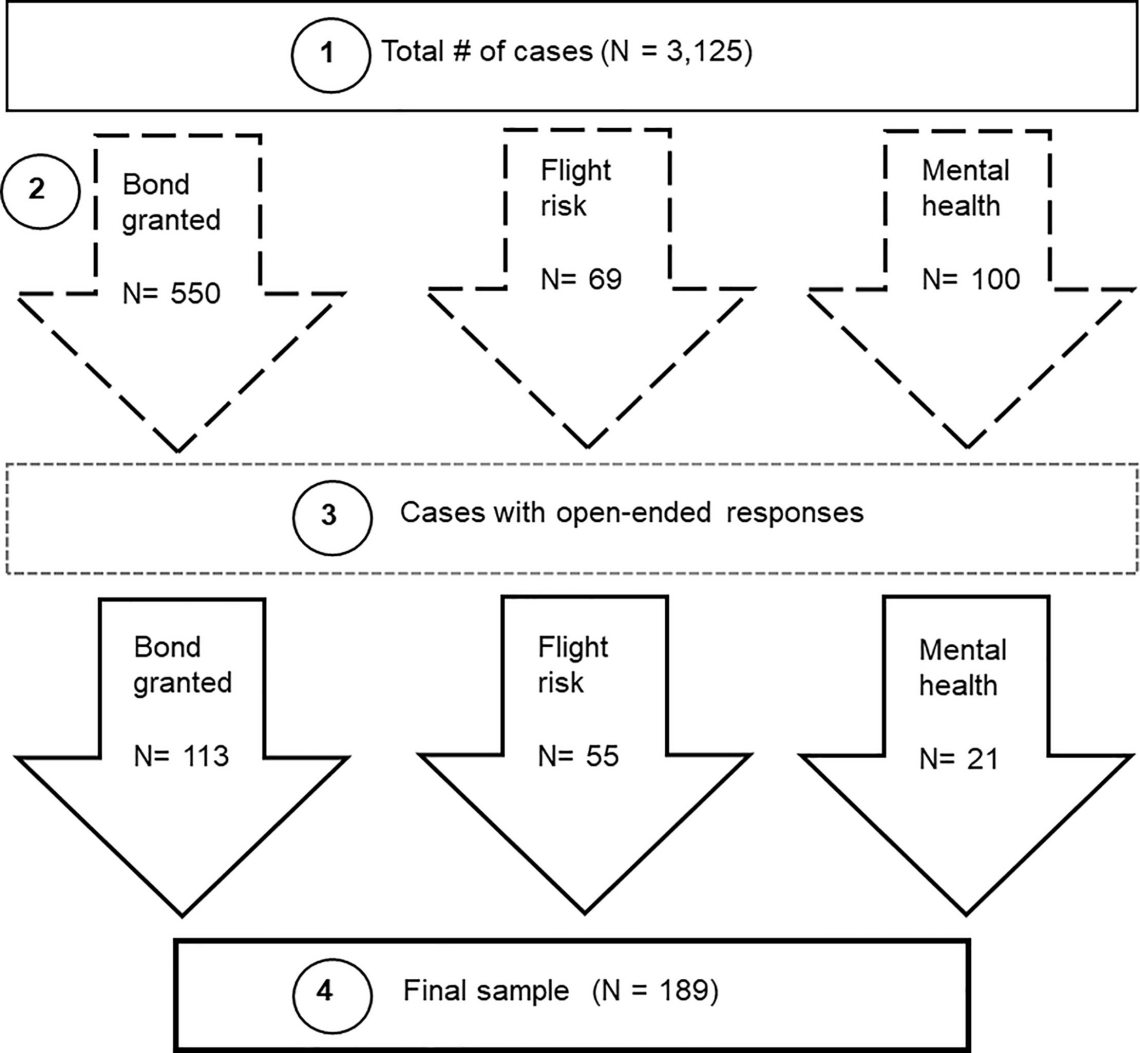
Framework for open ended data extraction process.

A total of 3,125 observations (step 1) of immigration case proceedings were conducted from July 2018 to June 2019 across 168 immigration court volunteers (Fig 1). Using these data, the purpose of the current study is to understand and highlight key perceived structural vulnerabilities experienced by immigrants who were undergoing immigration detention.

From the total sample, we selected cases (step 2) with affirmative responses to three questions: 1) whether detainees were granted bond (N = 550), 2) were deemed a flight risk (N = 69), 3) or had a reported mental health condition during the case (N = 100). Based on the overall length of cases, most lasting 5–10 minutes, the majority of cases did not have *any* responses recorded for the open-ended questions on the form or had very limited notes (e.g., few words), which were not included in the analysis. Duplicate cases were omitted in the final sample. Justification for extracting these cases is as follows: a bond being granted is a favorable outcome for the detainee in being released from immigration detention (as long as detainees can pay the bond) as they are also less likely to be deported, juxtaposed with being considered a flight risk, which hinders the detainees’ ability to obtain bond and be released from detention. Any reported mental health condition is also a critical source of vulnerability. Those with mental health conditions may be particularly vulnerable to factors and conditions specific to living in the US, including the effects of local immigration policies, discrimination and other stressors, frequency of relocation, and experiencing detention [8, 36], which may further worsen their overall mental health.

Next, two team members from the current study project reviewed all the cases for responses to the open-ended questions in the form (step 3) for cases that fit the criteria above. Of the cases that included open-ended responses, two team members reviewed each case and determined whether the responses could be thematically coded for perceived structural vulnerabilities based on previous literature using both deductive and inductive approaches for analyzing the data [37]. In using a deductive approach, team members discussed “a series of concepts, ideas, or topics” regarding vulnerability for coding and interpreting the data [37]. Team members met to discuss concepts and domains regarding structural vulnerability that have been previously highlighted in the literature. These domains were reflective of those found in Bourgois, Sue, Holmes, and Quesada’s [6] structural vulnerability assessment tool, which identified eight domains of vulnerability: financial security, residence, risk environments, food access, social network, legal status, education, and discrimination. Additionally, we drew from Cartwright’s [12] three components of “structures” of vulnerability, which include: 1) challenges, delays, or denials within the US immigration system, 2) challenges in securing steady employment at a living wage, and 3) lack of access to adequate health care including health insurance. These conceptualizations and domains of perceived structural vulnerabilities were used to determine the inclusion sample, as well as for coding and development of the codebook. The inductive approach to the analysis was determined by the content that emerged from the data [37] and provided more nuanced understanding of perceived vulnerabilities including navigating immigration detention and the immigration legal system. We followed a multi-step process for the inclusion of cases for coding (see below).

As part of the initial analysis, open-ended responses that pertained to concepts and domains of structural vulnerability as discussed above were extracted and transferred into word processing documents to thematically code. Any discrepancies in agreement regarding each case were brought up and discussed with the research team and during team meetings. A total of 189 cases were included in the analysis using notes across 82 immigration court observers (step 4), which represented about half (49%) of the total number of court observers (N = 186). On average, this sample of immigration court observers had attended and observed 30 immigration court cases. Please refer to the report by the Advocates for Human Rights [25] for further details regarding immigration court protocols and information on observers.

### Coding

For the open-ended responses, at least two members of the research team thematically coded each case. A codebook (available upon request) was established to document emerging themes centered on structural vulnerability and additional information (e.g., gender, legal representation, country of origin, primary language). The emerging codes were discussed among team members and during team meetings to ensure clarity and to establish consensus for finalizing the codebook. Two team members verified all the codes for all the cases. Any disagreement in codes was discussed across team members and coders to establish consensus and agreement in the codes. After all the cases were coded and verified, the data were uploaded into NVivo 12 Pro. NVivo software was used for data management and interpreting the data. Based on this analysis, we identified themes, domains, and examples of perceived structural vulnerabilities experienced by noncitizens attending case hearings in Bloomington, Minnesota.

## Results

The analysis included 189 cases. The majority of the sample was male (88%) and had legal representation or an attorney (61.4%). They also came from across 27 countries, with the majority from Mexico (37.6%) and other countries in Latin America (35.4%), followed by those from Europe, Africa, Asia, and Southeast Asia (19.5%). Due to the limited number of cases/detainees from other countries, these countries were collapsed into regions for descriptive purposes only. Additionally, the majority of this sample were not fluent in English, either speaking Spanish (65.1%) or another language (16.4%).

The results from this study are organized in a manner to reflect key areas of perceived structural vulnerabilities that are experienced by those held in immigration detention centers near the Bloomington, Minnesota immigration court. Three larger themes of perceived structural vulnerabilities emerged from this study. First, migration and motivations to migrate were a source of vulnerability. Second, general domains of perceived structural vulnerabilities that were outlined previously in the literature [6, 12] and that were also found among the sample of detained noncitizens in the current study. Third, given that these noncitizens were held in immigration detention centers, perceived structural vulnerabilities specific to undergoing immigration detention were identified.

### Migration

In this analysis, we found HRDP observers noting prior perceived vulnerabilities experienced by detainees in their home countries, in addition to vulnerabilities experienced through the process of migrating to the US. There were several examples of detainees’ previous experiences of trauma and fear of returning to their home country. Observers also noted the unsafe conditions and treatment that detainees experienced back in their home country.

“The detainee was claiming fear from government party as [they were] attacked

several times and tried to relocate but was followed…” (male, Bulgaria)

“Severely beaten by the police in [their] home country…” (male, unknown)

Another observer noted the conditions of abuse from which a detainee fled from their home country and left their children behind.

“The respondent fled for domestic abuse/childhood marriage via South Africa. Her sister is in South Africa. She has two children still in South Africa or ZI. The respondent had a hard time understanding the legal procedure and the right to appeal” (female, unknown).

Additionally, some detainees had documentation taken away from them prior to migrating to the US and were smuggled across the border.

“No I.D. with [them] at the border. Respondent states that passport was taken away from [them] in Mexico. Respondent’s attorney did have a copy of the passport” (male, China).

“Detainee [paid] $50,000 to a smuggler to get into US (he came in a trunk)—judge said detainee has means, so set bond [to $15,000]…” (male, China).

In summary, these examples illustrate vulnerabilities related to migration and detainees’ motivations to migrate ranging across unsafe living conditions in their country of origin, being smuggled into the US, and having their documentation taken away from them prior to migrating to the US. These experiences may have long lasting effects for their health and well-being as they may be traumatic in nature and lead to additional sources of vulnerability post migration.

### Structural vulnerability domains

Previous scholars identified general domains of structural vulnerabilities experienced by marginalized groups [6]. We adapted these domains from the themes that emerged from the present study, which are presented in Table 1. These domains include discrimination, financial insecurity (sub theme: challenges in paying for bond), social ties and family support, stable or fixed residence, English language proficiency (sub theme: interpreter challenges), and health and mental health (sub theme: access to medication). It is critical to note that because the individuals in the current study were detained, in addition to these broader systemic vulnerabilities, they were also experiencing vulnerabilities undergirded by immigration detention.

**Table 1:** Description and examples of perceived structural vulnerability domains.

Domain	Description	Example
**Discrimination**	Unclear reasons/ rationale for why ICE was called	“The respondent, from Zimbabwe, was driving [their] vehicle and was stopped for snow blocking his plates. The State Patrol who stopped him called ICE…[Their] attorney…argued that [they] should not be in detention due to the asylum application and that [they] should be released on [their] own recognizance. The judge seemed puzzled by why the respondent was in detention” (male, Zimbabwe)
**Financial insecurity**	Main source of financial support for family (spouse and children) and members with serious health conditions (e.g., children with cancer, disability), single parent, could not pay bond amount (even with family support), without proper documentation unable to obtain work permits so works odd jobs (e.g., landscaping, roofing)	“The detainee is a single parent of [their] son” (male, Mexico) “[Detainee] doesn’t have appropriate documentation to get work permit, so does odd jobs for cash (roofing, landscaping)…” (male, El Salvador)
***Subtheme*: *Challenges in paying for bond***	*References were made about detainees’ remaining in custody due to inability to pay for bond*, *challenges in paying for bond*, *or that bond was not lowered without a lawyer*	*“Detainee has not been able to secure the funds for bond*. *Remains in custody” (male*, *Mexico)* *“When the detainee was told [that the bond] had been increased [to $20*,*000]…[they] broke down emotionally*, *saying through the translator that [their] father was dead and [their] mother would have no way to come up with that amount” (male*, *Bulgaria)*.
**Social ties and family support**	Lack of family members in the US or support (e.g., letters) adversely affects ability to obtain bond (e.g., considered to be a flight risk)	“…flight risk for no family ties” (male, Mexico). “Respondent does not have any "proof" like letters from family. Judge states respondent lacks burden of proof [for considering bond]” (male, Honduras).
**Stable or fixed residence**	Reference to not having a stable or fixed address led to heightened concerns or being considered a flight risk or danger to society	“[Detainee] asked for bond to be lowered, but it was not granted as [they] seems to be a flight risk from [their] history of moving…” (male, Mexico) “Govt attorney stated [detainee] is a danger and that shouldn’t get bond because [they] didn’t know [their] own address…” (male, Honduras)
**English language proficiency**	Reference to challenges or inability to fill out forms in English and securing an attorney due to limited English abilities	“Detainee stated [they] couldn’t fill out forms in English as [they don’t] read or write…” (male, Mexico) “Since [detainee] can neither speak nor read English [they have] not been able to find an attorney” (female, Burundi)
***Subtheme*: *Interpreter challenges***	*Challenges were noted in having interpreters available and navigating immigration court even with an interpreter present*	*“Both agencies contacted*, *no Indonesian interpreter available…” (male*, *Indonesia)* *“Language Barriers- detainee is not understanding (even through interpreter) what judge is saying & asking for” (male*, *Guatemala)*
**Health and mental health**	Reference to detainees’ array of serious physical and mental health conditions which require access to health care, medication, and services	“[Detainee] takes insulin for diabetes since 28…” (male, Guatemala) “[Detainee]…suffers from PTSD, traumatic brain injury, depression, panic attacks” (female, Somalia) “Detainee has schizophrenia” (male, El Salvador)
***Subtheme*: *Access to medication***	*Reference to detainees not having access to needed medication*, *financial insecurity and lack of access to medication*	*“Difficult case*, *respondent requested removal because [they were] not being given [their] HIV medications while in detention*. *[They] said that [they] need to be deported to protect [their] health and well-being” (male*, *Palestine)*. *“Judge worried failure of detainee to receive medical injection would hamper ability to understand proceedings…” (male*, *El Salvador)*

#### Discrimination

HRDP observers noted unclear reasons for why ICE was originally called, which resulted in respondents being brought to immigration detention.

“The respondent, from Zimbabwe, was driving [their] vehicle and was stopped for snow blocking his plates. The State Patrol who stopped him called ICE…[Their] attorney…argued that [they] should not be in detention due to the asylum application and that [they] should be released on [their] own recognizance. The judge seemed puzzled by why the respondent was in detention” (male, Zimbabwe)

“[They were] arrested in a hotel room for "being noisy" even though [they were] in the room by [them]self…The hotel called ICE. The attorney mentioned that the hotel was known for calling ICE on suspected undocumented guests” (male, Spain)

Together, these examples illustrate how racial profiling may have contributed to ICE being called by the state trooper and hotel employees. As a result, detainees were apprehended and subsequently held in detention. These examples also demonstrate the complex nature of structural vulnerabilities that involves intersections of detainee’s precarious legal status and discrimination.

#### Financial insecurity

Many detainees were the main source of financial support for their family, often as sole providers. Financial responsibilities raised in hearings included supporting parents and children with serious health conditions, pregnant partners or young or newborn children. They also financially supported their partners and children with serious health conditions. Some detainees also had pregnant partners or newborn children.

“[Detainee] is sole provider for his children…” (male, Guatemala)

“The respondent’s attorney requested to lower bond…because of the respondent’s five US citizen children and the financial difficulty.” (female, Mexico)

“The detainee is a single parent of his son” (male, Mexico)

“…[their] child is going through cancer treatment” (male, Guatemala)

Prior to detention, detainees with a precarious legal status were unable to obtain work permits which resulted in limited employment opportunities. For this reason, detainees took on odd jobs that lacked security.

“[Detainee] doesn’t have appropriate documentation to get work permit, so does odd jobs for cash (roofing, landscaping)…” (male, El Salvador)

These examples demonstrate some of the financial vulnerabilities experienced by detained immigrants related to familial obligations and legal status-related employment constraints. Prior to detention, noncitizens were more likely to have low wage jobs and work in poor working conditions [19]. Detainees were often the head of their households and in financially challenging positions supporting their dependents while relying on informal work contracts necessitated by their lack of employment authorization.

#### Challenges in paying for bond (subtheme)

HRDP observers also noted the challenges detainees faced in paying for their bond even with support from their family. For those who were granted bond and could not afford bond, they continued to remain in custody.

“Detainee has not been able to secure the funds for bond. Remains in custody” (male, Mexico)

Another detainee became distraught when, despite being granted bond, they shared that it was not possible for them to pay for the set bond amount.

“When the detainee was told [that the bond] had been increased [to $20,000]…[they] broke down emotionally, saying through the translator that [their] father was dead and [their] mother would have no way to come up with that amount” (male, Bulgaria)

In describing why one detainee was unable to obtain bond, it was noted that they requested to have their bond lowered; however, the immigration judge stated that they needed to obtain a lawyer to reduce their bond amount.

“Bond was not granted. Pro se [or without representation] detainee requested to lower $5K bond…Request to lower bond needs to be in writing describing why bond needs to be lowered. Was given list of free/affordable attorneys to request bond help” (female, Mexico)

These examples demonstrate the complexity of financial challenges experienced by detainees. In these specific examples, though the detainees were granted bond, they experienced further challenges in being able to pay for their bond, which contributed to constrained options. Additionally, detainees without legal representation were unable to negotiate a lower bond.

#### Social ties and family support

Detainees without social ties in the US, especially those without family in the US, were viewed more negatively than detainees who could summon support from US social ties. Lacking evidence of family and community support in the form of letters adversely affected bond decisions.

“Respondent does not have any "proof" like letters from family. Judge states respondent lacks burden of proof [for considering bond]” (male, Honduras)

“…flight risk for no family ties…” (male, Mexico)

Some immigration judges perceived detainees without social ties as suggestive of being a greater “flight risk” or that they would be less likely to show up in court.

“No immediate relatives in US, concern that detainee will show up for court…” (female, Somalia

In sum, these examples show that not having family members in the US may negatively impact bond outcomes, and in turn, may further lengthen the process of being held in detention.

#### Stable or fixed residence

HRDP observers recorded several cases in which detainees did not have a stable or fixed address, had a history of moving, or did not know their address. In these instances, lack of stable residence appeared to negatively affect bond outcomes as the immigration judge expressed concerns about the detainee being a flight risk or danger to society.

“[Detainee] asked for bond to be lowered, but it was not granted as [they] seem to be a flight risk from [their] history of moving…” (male, Mexico)

“In the past, the [detainee] couldn’t provide an address of residence, which was a concern of the judge” (male, Ecuador)

“Govt attorney stated [detainee] is a danger and that [they] shouldn’t get bond because [they] didn’t know [their] own address…” (male, Honduras)

Noncitizens face challenges in obtaining adequate housing due to their legal status and marginalization (living in poverty, low wage work, etc.). Frequent moving and the lack of a stable residence appeared to influence the immigration judges’ decisions in granting bond, as these detainees were perceived as a flight risk or danger. These examples demonstrate the intersecting vulnerabilities that emerge across housing and immigration detention for detained noncitizens.

#### English language proficiency

Detainees with limited English language proficiency experienced challenges in being able to fill out necessary documents in English. Furthermore, this also affected their ability to contact or secure an attorney.

“Detainee stated [they] couldn’t fill out forms in English as [they don’t] read or write…” (male, Mexico)

“Since [detainee] can neither speak nor read English [they have] not been able to find an attorney” (female, Burundi)

“Detainee stated [they] couldn’t fill out forms in English as [they don’t] read or write and couldn’t contact a lawyer from jail” (male, Mexico)

An observer noted that the detainee didn’t know what the role of an attorney was, in addition to highlighting challenges around filling out necessary paperwork with their limited English abilities.

“The detainee did not have an attorney and appeared to be uncertain as to what the role of an attorney is. She was also unsure as to how complete the documents if she doesn’t have anyone to help her and does not speak English. She does fear she may be in danger if she went back” (female, Mexico)

Together, these examples demonstrate that detainees without a strong command of English experienced further challenges, including being able to fill out necessary forms and obtain a lawyer, which has profound consequences in navigating immigration detention.

#### Interpreter challenges (subtheme)

References were made regarding challenges around finding interpreters for specific languages and dialects (e.g., Hmong, Punjabi, Indonesian).

“Both agencies contacted, no Indonesian interpreter available…” (male, Indonesia)

“Hmong interpreter not available, tried again in 15 minutes, then attempted to schedule interpreter in 30 minutes; 2 tries on 2nd attempt…” (male, Laos)

Even with an interpreter present, there were noted challenges in understanding immigration court proceedings, along with specific portions of the case directly interpreted.

“Language Barriers- detainee is not understanding (even through interpreter) what judge is saying & asking for” (male, Guatemala)

“Interpreter only interpreted the questions and comments directed to respondent, not any of the attorney’s statements…” (male, Guatemala)

There were also technology and connectivity issues noted during the proceedings, specifically with regards to having both the attorney and translator both join via phone.

“Technology didn’t allow attorney and translator to speak on the phone at the same time…” (male, Bulgaria)

These examples demonstrate that there were no interpreters available for some languages, leaving detainees without the ability to understand what is occuring during their case hearing. Additionally, even with an interpreter, there are a multitude of challenges including the actual translation of the information to the detainee and communication technology issues. These issues are exacerbated when the detainee is not represented, as they are left not only to navigate the courtroom in a foreign language, but without the necessary support to understand the legal jargon as well.

#### Health and mental health

In some cases, HRDP observers noted detainees’ general health and mental health conditions.

“He takes insulin for diabetes since 28…” (male, Guatemala)

“[Detainee]…suffers from PTSD, traumatic brain injury, depression, panic attacks” (female, Somalia)

“Detainee has schizophrenia” (male, El Salvador)

There were also noted cases of mental health worsening while being detained.

“[Detainee] was on ‘suicide watch’ not clear which jail and 14 days monitoring…” (male, Mexico)

Based on these examples, some detainees are experiencing an array of serious health and mental health conditions where it is imperative that they have access to health care, medication, and services. Ongoing detention is especially a threat for these individuals and may exacerbate their health and mental health, as well as contribute to further marginalization (e.g., trauma).

#### Access to medication (subtheme)

Observers noted that some detainees did not have access to their medication. Without their medication, an immigration judge expressed their concern for the detainee in navigating immigration proceedings.

“Judge worried failure of detainee to receive medical injection would hamper ability to understand proceedings…” (male, El Salvador)

Another detainee requested to be removed due to not having access to HIV medication while in immigration detention to protect their health.

“Difficult case, respondent requested removal because [they were] not being given [their] HIV medications while in detention. [They] said that [they] need to be deported to protect [their] health and well-being” (male, Palestine)

Previous experiences of financial hardship in being able to pay for insulin were also noted.

“He borrowed money from someone to pay for his insulin, and when the detainee couldn’t pay, the person threatened the detainee” (male, Guatemala)

To summarize, some detainees are experiencing serious mental and physical health conditions which require treatment, and without access to medication, this profoundly affects their ability to navigate immigration proceedings.

### Immigration detention

Cartwright [12] argued that the US immigration system creates vulnerabilities for immigrants via challenges, delays, and denials. This observation is also supported in the current analysis for noncitizens undergoing detention. Challenges faced by these detainees include navigating immigration court proceedings, securing representation, lengthy administrative processing times, and obtaining necessary information for their case while in detention.

“Respondent’s attorney was for bond only. Requested continuance to give respondent time to find an attorney for his removal hearing” (male, Ecuador)

“Detainee has asked several times for an extension and has not been able to find an attorney” (male, Honduras)

“U-Visa pending for 4 years already” (male, Mexico)

“Bond granted [$4,000]..Has been waiting for asylum interview for 3 years…” (male, unknown)

“Plead it is difficult to get information/ documents about what happened in Nicaragua while detained” (male, Nicaragua)

To further illustrate, the detainee needed help navigating his case and had not been aware that they had been granted bond, further illustrating the complexity of challenges detainees are experiencing. The detainee was also ill and needed help; however, he did not know how to request help.

“Respondent appeared confused by the proceedings. He said he has pneumonia and unaware of how to ask for help. He said his fiancée is ill and needs help. He also asked for bond and was unaware that it had been granted. He appeared stressed and unsure of his options. He needs help to navigate the court system” (male, Honduras)

In another case, the detainee could not understand the legal process no matter how many times the judge tried to provide further explanations. This eventually led to the detainee giving up and requesting to be sent back home, or voluntary departure.

“The hearing took 5 minutes because the detainee didn’t understand the process and when…[the immigration judge]… explained it, he used the formal words of the court and the [detainee] didn’t understand "appeal." They went around and around. The [detainee] said many times that it was ‘Ok’ and he just wanted to go back to Mexico…” (male, Mexico)

In addition to the challenges of navigating hearings, there were several references to mistreatment while in detention.

“At [respondent’s] urging, ‘I haven’t been treated well, by being moved fast from Freeborn to Sherburne, put immediately into 5 days of solitary (SOP)’…and his lack of getting meds since put in ICE custody a month ago" (male, Mexico)

“Detainee showed his Attorney how tight his shackles were around his ankles, he has bruising from the continued restraint” (male, Honduras)

Lastly, there were references to the stress associated with being held in detention as well as the loss of hope.

“Respondent seemed to have [their] mind made up going into the hearing; that [their] situation was hopeless” (male, Honduras)

“Detainee did not want to sit in custody and asked to be removed to Mexico” (male, Mexico)

These examples provide compelling evidence of perceived vulnerabilities that stem from immigration detention. Several cases illustrate detainees facing numerous challenges in navigating detention and the cascading vulnerabilities, contributing to feelings of frustration, hopelessness, and ultimately requests to be deported.

## Discussion

The purpose of the current study was to illuminate perceived vulnerabilities of noncitizens as they are raised and described during immigration court proceedings using the cases observed at the Bloomington, Minnesota court. Through a thematic analysis using notes from immigration court observers, three main themes of perceived vulnerability were identified. These themes include: 1) migration and motivations to migrate, 2) perceived structural vulnerabilities experienced while living in the US, and 3), challenges in navigating immigration detention. Collectively, these findings demonstrate that noncitizens who were undergoing immigration detention may experience multiple, intersecting vulnerabilities. These vulnerabilities may also impact their families, especially since many detainees were supporting family members including pregnant partners, children, and those with chronic health conditions. A growing number of studies have found the harmful effects of anti-immigrant stigma and deportation on health and integration for immigrants and their families [38–40]. However, the findings of the current study provide a novel contribution in understanding perceived structural vulnerabilities that were discussed during immigration court hearings among noncitizens facing detention and possible deportation.

The first theme captured perceived vulnerabilities pertaining to the detainees’ previous conditions and experiences in their home country or through the process of migrating to the US. Some detainees expressed trauma and fear of return to their home country due to unsafe conditions. Li [41] found that pre-migration experiences of trauma have lingering effects for post migration integration including legal status stressors, social isolation, and discrimination among Asian and Latinx immigrants. Additionally, the results of the current study show that some detainees had their documentation taken away from them at the border or prior to their arrival to the US. Without any identification, individuals will experience additional challenges in securing proper documentation and identification for entering a new country, as well as adversities in integration. The research literature has primarily focused on undocumented status or entering without legal documentation, however limited attention has been paid to understanding the implications of the individual missing any forms of identification or documentation as a source of vulnerability. These findings provide evidence to support the notion that migration is a critical determinant of vulnerability [13], which has deleterious consequences for integration.

The second theme focused on perceived structural vulnerabilities which have been previously identified in the literature among marginalized populations [6]. These domains included experiences of discrimination, financial insecurity, social support and ties, English proficiency, and health. We find that these domains were also evident in the sample of the current study of noncitizens undergoing detention. Morey [38] argues that citizenship status is a concealed form of identity and with the rise of anti-immigrant stigma over the past decade, being an immigrant has been conflated with undocumented status. Further, racial/ethnic minorities who might be suspected of being an immigrant may be victims of stigma or racial profiling, regardless of their legal status. We also find evidence that some detainees may have been victims of racial profiling and were brought into detention as a result.

Noncitizens experience marginalization in obtaining steady employment and livable wages and are also more likely to live in poverty [12, 18], with our study corroborating these findings. In our study, we also find that they are experiencing challenges regarding securing stable employment and livable wages. Some also took on odd jobs prior to detention as they are unable to obtain secure work permits due to their legal status. Our findings also suggest that financial insecurity has profound implications for detainees and their family (e.g., sole providers) and other family members with chronic and serious medical conditions. The loss of an income earner or caregiver leads to even more constrained financial situations including housing and food insecurity [42]. The financial insecurity among detainees in the current study was further heightened by receiving bond amounts that were beyond their ability to pay, which aligns with previous research [1, 23]. Lacking sufficient funds to pay for bond contributes to prolonged separation from family and further exacerbates challenges in being released from detention [1, 24].

Additional domains of perceived vulnerabilities included limited social ties in the US, not having a fixed or stable address, limited English language proficiency, and health and mental health conditions. These vulnerabilities not only stemmed from the detainees’ immigration status, however, due to the intersecting nature of these vulnerabilities, this also in turn, impacted bond outcomes. Specifically, if detainees didn’t have a fixed address or had limited social ties in the US, they were then perceived negatively as a flight risk or a danger to society in immigration court, and thus, contributing to adverse consequences in obtaining bond. Noncitizens are more likely to experience challenges in obtaining stable and adequate housing and are less likely to be homeowners [43].

Further, many of these detainees have limited command of English and face further challenges in navigating immigration detention and immigration court proceedings (e.g., filling out forms in English, securing representation). Reports show that immigrants while in detention do not have adequate assistance for securing legal counsel, translation or interpretation services [24]. Representation has been associated with contributing to better outcomes including securing bond and relief from deportation [23, 28]. However, due to their limited English language proficiency, it appears to further place detainees in a vulnerable situation.

Additionally, there were noted instances where the immigration judge was concerned about the detainee in navigating immigration proceedings without access to their medication for those with serious health and mental health conditions. For detainees, the increase in distress due to not having access to medication led to requests to be removed. In summary, the depth of vulnerability experienced by detainees is further heightened by limited social ties and English language proficiency and inadequate access to housing and health care, which has profound implications for obtaining bond as well as their overall health.

The third theme reflects the challenges detainees experience in undergoing immigration detention and navigating the US immigration legal system, specifically, the difficulties, delays, and denials, as previously outlined in prior research [12]. Detainees expressed challenges and distress in navigating the immigration court process, finding and securing representation, and delays in obtaining a visa and interview. Several detainees also expressed mistreatment, the loss of hope, and the inability to continue to stay in detention. There has also been increasing reports of the poor conditions of immigrant detention centers including physical restraints and lack of access to health care, and mistreatment from detention center officers [24] and in most extreme cases, death [44]. The uncertainty of release among detained immigrants generates extreme stress [32], as many are detained for months or even years [24]. Navigating immigration detention is complex and challenging, undergirded by distress generated from this experience, which has lasting and profound implications beyond their release from detention.

**Fig 2:**
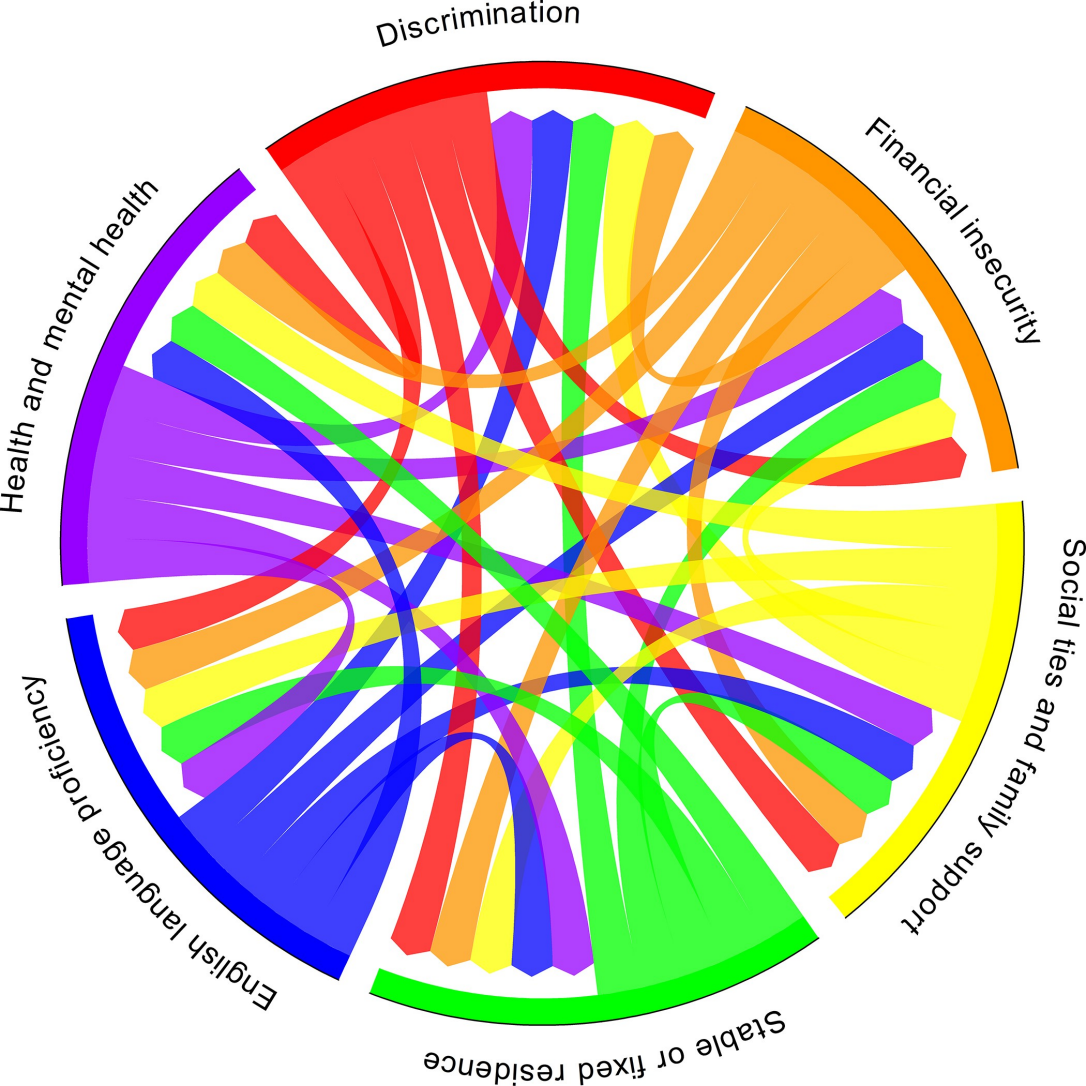
Intersecting perceived structural vulnerabilities.

An emerging area of research has begun to discuss multiple and concurrent adversities or sources of vulnerability, otherwise known as syndemic theory [8]. Though the nature of the data in the current study did not allow us to test syndemic theory as previously done in empirical research [45], we did find that some of these domains appear to intersect with one another, which is consistent with potential syndemic relationships. Fig 2 visually depicts these intersections across each of the structural vulnerabilities as identified in the current study. For example, legal or noncitizen status was noted to influence other domains, including experiences of constrained employment options and discrimination, which, in turn, led to being apprehended by ICE. Additionally, financial insecurity affected multiple domains of vulnerabilities, including the detainees’ inability to financially support their family, access necessary medication, or obtain a lawyer and ultimately, not being able to afford their bond. Detainees with limited English proficiency experienced further challenges in obtaining a lawyer or filling out necessary forms in English and also navigating their own case in immigration court. In addition to the challenges in navigating immigration proceedings, these detainees were often alone without any support from family or friends. These factors coupled with experiencing multiple intersecting forms of adversity may further heighten their existing vulnerabilities. For instance, one detainee expressed confusion regarding the immigration proceedings and didn’t realize their bond was granted, while at the same time had pneumonia and experienced challenges supporting their fiancée who was also ill. This example, along with the other results from the current study, also support mechanisms as outlined in the Theory of Fundamental Causes, specifically financial insecurity, and limited knowledge and support from family and friends deleteriously impact immigration proceedings and bond outcomes for noncitizens undergoing detention. There has been increasing evidence which demonstrates the harmful consequences of immigration detention and deportation for the health and mental health of US citizen family members, including children of those who have been detained or deported, illustrating the spillover effects of detention on families [1, 34, 38, 46]. The findings of the current study highlight the complexities and multiple intersections of perceived vulnerabilities experienced by noncitizen immigrants under restrictive immigration surveillance.

The sample of noncitizens in the current study experienced vulnerabilities due to the precarity of their legal status along with immigration detention, calling for the need to consider systemic solutions in rethinking the immigration system in the US. We recognize that systemic change may take many years, and thus, policies which focus on contemporary issues may curb some of these adverse effects faced by detained noncitizens; therefore, more attention is needed on both short-term and long-term solutions. The results of the current study demonstrate support for policies and recommendations brought forth by the Advocates for Human Rights and other organizations [25, 47]. Their recommendations include eliminating a $1,500 minimum bond and for immigration judges to factor in the detainees’ ability to pay for decisions on bond outcomes. Additionally, they recommend a full functioning immigration court system which includes staff support and availability of interpreters (especially from Africa and Asian countries), providing reasonable timelines for filing applications and documents, training personnel to use plain language and not legal jargon, and accessibility and support for those with low literacy or limited English language proficiency. As evidenced by the disparities in outcomes among those with and without representation [28], it is critical to provide greater access to legal representation, including pro bono counsel and appropriate technology for communication methods with their lawyer and families. The Advocates for Human Rights [25] also recommend ending detention practices of routine shackling and solitary confinement to promote the human dignity of noncitizens immigrants. We support these recommendations based on the results of the current study. Furthermore, given some detainees had issues obtaining their medication, we also advocate for immigration detention centers to provide detainees with access to their medication while being held in detention.

Larger system-wide changes are needed in addition to the shorter-term proposed solutions. Longer term solutions involve moving towards comprehensive immigration reforms of providing permanent pathways to citizenship for noncitizen immigrants so that they have access to guaranteed full protections as a citizen [34]. The combination of both short-term and long-term efforts can tackle different aspects of the immigration system leading to sustained changes for noncitizens in the US.

Several limitations should be considered for the present study. First, these data may not be generalizable to all noncitizens experiencing detention, as circumstances and opportunities may differ by court dockets and geographic locations [48]. The analysis also relied on notes from self-selected third-party immigration court observers during master calendar hearings. The nature of the data does not capture the entirety of the detainees’ background and circumstances unless it was pertinent information mentioned relative to their case. Some detainees brought up some of the challenges they may be experiencing and were included in observer’s notes; however, the original data and purpose of the HRDP study was not focused on investigating vulnerability. Thus, detailed information regarding these experiences of vulnerability and other domains of vulnerability were not collected. Hence, the overall data reflect perceptions of vulnerability and other demographic information that was collected of the detainee (e.g., English knowledge, country of origin). More empirical research is needed to examine structural vulnerabilities among detained noncitizens and its spillover effects onto their families. It has also become increasingly vital to explore interactive and multiplicative relationships between vulnerabilities among detainees using quantitative methods. The majority of these hearings also tended to last 5–10 minutes, with cases often finishing at a rapid pace. It is critical to note the variability in the information recorded in the notes by court observers due to factors regarding their levels of understanding of immigration law (e.g., jargon), their rapid note taking abilities, or where they may be sitting in court (e.g., front, back). Nevertheless, the data used for the current study provide a unique and important insight into understanding the multidimensional nature of vulnerability experienced by noncitizens undergoing detention.

## Conclusions

The findings of our study raise concerns regarding the multitude of vulnerabilities experienced by detained noncitizens, as well as possible extensions of vulnerabilities to detainees’ US citizen family members. Our findings suggest multiple intersecting adversities across systems which may further lead to greater vulnerabilities. Immigrants should be treated with dignity and respect, regardless of their legal status. Comprehensive immigration reforms are necessary, including both short-term and long-term solutions and the enactment of policies which address pressing issues for noncitizens in navigating immigration detention. There is an increasing need for practitioners and scholars working in collaboration across fields and expertise in reforming the current immigration legal system and for enacting policies, practices, and laws which consider the dignity and respect of human life.
